# The Scale Analysis of Number Mapping onto Space: Manual Estimation Study

**DOI:** 10.1080/17470218.2013.782325

**Published:** 2013-12-01

**Authors:** Vyacheslav Karolis

**Affiliations:** Institute of Cognitive Neuroscience, University College London, London, UK

**Keywords:** Manual estimation, Scale, Number, Space, Shared metrics

## Abstract

Previous studies have shown an interference of task-irrelevant numerical information with the spatial parameters of visuomotor behaviour. These findings lend support to the notion that number and space share a common metric with respect to action. Here I argue that the demonstration of the structural similarity between scales for number and space would be a more stringent test for the shared metrics than a mere fact of interference. The present study investigated the scale of number mapping onto space in a manual estimation task. The physical size of target stimuli and the magnitudes of task-irrelevant numbers were parametrically manipulated in the context of the Titchener illusion. The results revealed different scaling schemas for number and space. Whereas estimates in response to changes in stimulus physical size showed a gradual increase, the effect of number was categorical with the largest number (9) showing greater manual estimate than the other numbers (1, 3, and 7). Possible interpretations that are not necessarily incompatible with the hypothesis of shared metrics with respect to action are proposed. However, the present findings suggest that a meticulous scale analysis is required in order to determine the nature of number–space interaction.

It is proposed that the representations of number, space, and time utilize a common magnitude system required to bring together magnitude information from different modalities in order to subsequently use it for visuomotor transformations (Bueti & Walsh, 2009; Walsh, 2003). This hypothesis is supported by two lines of evidence. First, neuroimaging and neurophysiological studies show that the representations of number, space, and time partially overlap in the parietal cortex (Pinel, Le Bihan, Piazza, & Dehaene, 2004; Sawamura, Shima, & Tanji, 2002; Simon, Mangin, Cohen, Le Bihan, & Dehaene, 2002; review: Hubbard, Piazza, Pinel, & Dehaene, 2005). Secondly, the studies of visuomotor tasks show that task-irrelevant numerical information may interact with the spatial parameters of motor response—for example, with the spatial path of reaching (Song & Nakayama, 2008) and with the magnitude of grip aperture (Andres, Ostry, Nicol, & Paus, 2008; Lindeman, Abolafia, Girardi, & Bekkering, 2007). These findings suggest that different magnitudes are related by the common metric for action and that “the parietal cortex transformations, that are often assumed to compute ‘*where*’ in the space, really answer the questions ‘*how far, how fast, how much, how long and how many’* in respect to action” (Walsh, 2003, p. 486, original italics).

Although this interference of number on the parameters of movement may seem to strongly support the hypothesis of a common metric for number and space with respect to action, it is important to note that spatial representations may occur in two complementary forms (Kosslyn, 1987; Logan, 1995). The first is a categorical form of representation that reflects the cognitive ability to conceptualize experience. Terms such as “extreme left”, “rightwards”, “centre”, and “top”, as directional markers, may play an important role in movement planning (cf. Glover, 2004) but they are not sufficient for guiding the limb to a precise location in space in order to, for example, grasp an object. To achieve the goal, the motor system requires fine-grained representations of space. The latter appear to better fit the notion of the metrics for action, but it remains unclear whether number affects specifically this form of representations and not the other. The rejection of an idea that number interacts with categorical system would be unwarranted given that support for the categorical mapping of number onto space is well documented outside the visuomotor domain (Tzelgov, Meyer, & Henik, 1992; Gevers, Verguts, Reynovoet, Caessens, & Fias, 2006). The situation in the visuomotor studies of number has not been helped by a frequent use of categorical experimental designs with extreme numerical magnitudes grouped as large and small (e.g., 1 and 2 vs. 8 and 9; e.g., Andres et al., 2008; Fischer, 2003; Lindeman et al., 2007). Such approach is hardly diagnostic for the type of number mapping onto space.

A more stringent criterion is therefore required to establish whether number and space share a common metric with respect to action. This may be derived from a formal description of metrics—that is, the scaling theory (Stevens, 1951). One can propose that the critical test for this hypothesis is to show a structural similarity in the scales for number and space, at least as they can be inferred from the observations of behavioural outcomes. For example, in grasping tasks, the gradual increase in the size of an object leads to the gradual increase of aperture (Marteniuk, Leavitt, MacKenzie, & Athenes, 1990). Consequently, if number and space share a fine-grained metric with respect to action, one should also expect a parametric effect of number on the parameters of movement. In other words, some value, proportional to task-irrelevant number magnitude, is expected to add up to a computed size of an object, resulting in a gradual modification of grasp aperture. The critical point is that without a demonstration of the structural similarity between scales for number and space one cannot tell whether the effect of number on visuomotor performance is determined by the common metric with respect to action or whether it represents a contextual bias similar to that shown for words with implicit magnitude semantics (e.g., Gentilucci, Benuzzi, Bertolani, Daprati, & Gangitano, 2000; Glover, Rosenbaum, Graham, & Dixon, 2004).

The parametric effects have been observed in two grasping studies where subjects were required to select between two types of motor response in the parity judgement task (opening/closing finger aperture—Andres, Davare, Pesenti, Olivier, & Seron, 2004; power/precision grip—Moretto & di Pellegrino, 2008). However, these studies do not provide a direct spatial measure for the effect of number and show a gradual effect on the latencies in the two-alternative forced choice of a response type. Interpretation of the interference with the selection between two response alternatives is not straightforward per se. Several authors argued that the interference of number with spatial processing occurs here from the competition between spatial and numerical codes at the response selection stage (Keus, Jenks, & Schwarz, 2005; Keus & Schwarz, 2005). Such competition may originate from an association of the verbal concepts applied to number and space, also known as polarity coding (e.g., small/left vs. large/right; e.g., Gevers et al., 2010; Proctor & Cho, 2006).

Consequently, a better test would be to show that the parametric effect occurs within one type of motor behaviour. Although there is limited evidence for a parametric effect of number on the spatial path of movement, showing the association between number and location (Song & Nakayama, 2008; but see Santens, Goossens, & Verguts, 2011), the effect of number on the parameters of grasping, which could indicate an association between number and spatial magnitude, does not always conform to the metrics-for-action hypothesis. For example, Andres et al. (2008) found that the effect of number on the grasp aperture is greater when subjects reach for a larger object. In contrast, the maximum grip aperture has been shown to be a linear function of an object's size (Marteniuk et al., 1990). In other words, the effect of the spatial magnitude on the aperture is additive whereas the effect of number is multiplicative, or exponential-like. This suggests a structural dissimilarity of the scales for number and space.

In this study, the scale of numerical mapping onto space was investigated using a manual estimation task whereby subjects were required to provide a report about perceived stimulus magnitude by scaling the distance between the index finger and the thumb, also known as aperture (Amazeen & DaSilva, 2005; Haffenden & Goodale, 1998). Under normal circumstances, a manual estimation is not restricted by time, and the desired precision may be achieved using proprioceptive and/or visual feedback. This would allow one to test whether the effect of numerical information on the spatial parameters of movement is short-lived. An example of this can be seen in Andres et al. (2008) who found that number interferes at the early stage of movement execution and who argued that control mechanisms may counteract the interference of number magnitude in later stages to allow a precise scaling in accordance with actual object size (also see Glover et al., 2004).

In the present study, manual estimates were provided in the context of the Titchener illusion. The display for this illusion contained a target circle surrounded by an array of circles that were either small or large. A target surrounded by larger circles is generally perceived as being smaller than an identical target surrounded by smaller circles. Additionally, four levels of numerical magnitude (1, 3, 7, 9) presented inside the target circle were used. The trials with no number presented were also included to discourage subjects from thinking that presentation of a number may somehow relate to the purpose of the study.

Given that the study was concerned with fine-grained parametric effects, the critical issue was whether manual estimates could veridically differentiate between relatively fine-grained differences in the stimuli. Previous studies of manual estimation do not report how the estimates change with small parametric increases in stimulus magnitude. It is possible that responding in the task is categorical and renders roughly big estimates if the stimulus is perceived as big and renders roughly small estimates otherwise. To obtain a reliable evidence for fine-grained estimations, five levels of target size with 1-mm step between two adjacent levels were used.

The study comprised two experiments in two independent groups of subjects with the only difference being that subjects in Experiment 1 responded without seeing their hand (open-loop, OL, condition), whereas in Experiment 2 the visual feedback was available (closed-loop, CL, condition). This manipulation was used since motor responses may be less affected by contextual information in the presence of the sensory feedback (Bruno & Franz, 2009; Glover, 2004), and consequently, a significant effect in one feedback condition may not necessary generalize to the other.

## Method

### Subjects

Healthy adult subjects were recruited via the University College London (UCL) subject pool and gave informed consent to participate. They were remunerated for their participation. All subjects reported to be right-handed and had normal or corrected-to-normal vision. Twenty subjects were tested in each experiment (open-loop experiment: 9 male, mean age *=* 25.4 years, *SD =* 4.9; closed-loop experiment: 10 male, mean age *=* 22.7 years, *SD =* 5.1).

### Apparatus

**Figure 1.**
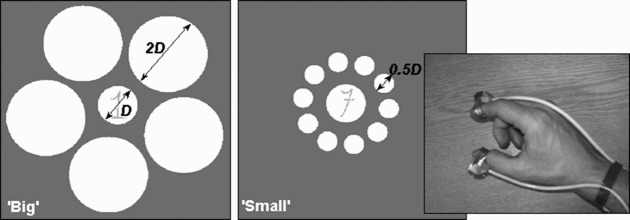
Stimulus material (left and centre picture) and response (right picture). The letter “D” stands for the diameter of the target circle. Five sizes of D at 1.02-mm steps, starting from 30.6, were used. The diameters for circles in the big (on the left) and small arrays (in the centre) were 2D and 0.5D, respectively. Numerical symbols (1, 3, 7, or 9) in the middle of the target circle contained the same number of pixels for each size of the target. The responses were collected using a motion-tracking device. The sensors were taped to the distal phalanges of the index finger and thumb. The trial always started with fingers pinched together (zero-distance aperture). In the open-loop condition, the hand was placed in a nontransparent box, preventing the sight of the hand.

The experiment was conducted in a darkened room with head movements of subjects restricted by a chinrest located 570 mm in front of the 20.1″ LCD monitor (1,600 × 1,200 pixels, pixel size 0.255 mm). The midline of the eyesight approximately coincided with the centre of the monitor. In one of the feedback conditions (open-loop, see below) subjects were instructed to keep their right hand in an opaque box (200 × 200 × 150 mm). A motion-tracking Fastrak 3Space system (Polhemus Inc.) with a sampling rate of 120 Hz and spatial static accuracy of 0.8 mm was used to collect kinematic data. Two sensors were taped on the top of the most distal phalanges of the index finger and the thumb (Figure 1).

### Stimuli and design

The experiment was administered in two independent groups. In the OL group, subjects kept their right hand in an opaque box and were unable to monitor their responses visually. In the CL group, subjects held their hand in front of their body so that their experimental hand was within vision.

The stimulus display showed a target circle surrounded by an array of nonoverlapping white circles (Figure 1) presented against a grey background. The circles in the surrounding array were evenly distributed around the target circle with their centres equidistant from it. The angle for the centres of the circles in the array was varied pseudorandomly. The radius of the target circle was manipulated parametrically in steps of 1.02 mm (4 pixels). There were five sizes for the target circle with a minimum diameter of 30.6 mm and a maximum of 34.68 mm. The second experimental variable was the type of the surrounding circles, or *array*. In the big array, there were five circles surrounding the target circle with their radii twice as long as that of the target circle. In the small array, there were 10 circles surrounding the target circle with their radii half as long as that of the target circle. The distance between the rims of the middle circle and the circles in the array was fixed at 21.2 mm and was chosen to minimize variation in the distances between the rims of the circles in the surrounding array as they were changing as a function of the target size.

The third experimental variable was the *number* presented inside the target circle. The colour of a number was half-saturated grey. A number was one of four Arabic numerals (1, 3, 7, and 9) created on the basis of Bradley Hand ITC font. Numerical symbols were approximately equal in size (the height was equal to the radius of the target circle divided by 2, and the width was the radius divided by 4, at the highest and the widest points, respectively) and were composed of the same number of pixels for every size of the target circle (minimum, 1,800 pixels; maximum, 2,178 pixels, the difference in pixels for numbers presented in the circles of two adjacent sizes being 126 ± 4 pixels). In addition, the *no-number* condition, in which the target circle did not contain any number, was also presented. Its functional role was to prevent subjects from thinking that the experiment is “all about numbers”. The data for this condition were excluded from the analysis.

The design of 2 (array: big and small) × 5 (size of the target circle) × 5 (number: 1, 3, 7, 9 plus no-number condition) factors rendered 50 variable combinations. The experiment consisted of eight blocks of 50 trials; each condition was presented once within each block.

### Procedure

The procedure was self-paced with stimulus presentation controlled by the experimenter. The design, stimuli, apparatus, and procedure were identical in both experiments. The lighting conditions were also identical. The only source of light was the ambient light of the monitor, which was sufficiently bright to see the hand in the CL condition. The alignment of the aperture with the stimulus in order to make a direct comparison was not permitted in the CL condition. Here either the hand or the stimulus could be in the central visual field but not both.

At the beginning of each trial, subjects were presented with an empty grey screen and were instructed to close the aperture between index finger and thumb by the vocal instruction “pinch together” from the experimenter. Subjects were asked to do this in a natural and consistent way without squeezing their fingers together. The purpose of closing finger aperture was twofold. First, a reading from the sensors was made just before a stimulus presentation, determining a zero distance between fingers. Second, it resulted in a similarity of the initial state for each particular trial so that magnitude of response could be related to the amplitude of movement. After a subject pinched the fingers together, the stimulus was displayed on the screen. The subject was required to open up and scale the aperture between the index finger and the thumb such that it would match the size of the target circle. Once subjects decided that the aperture was appropriately scaled, they were required to give a vocal signal that they were ready. This vocal signal was either the identity of a number contained within the target or saying “none” in the no-number condition. On hearing the signal, the experimenter pressed a button to cause a white mask to cover the screen for 800 ms. During this time, subjects were required to keep their finger aperture “frozen”. After the white mask disappeared, the screen again turned grey, and the following trial was started. Subjects were allowed to rest between experimental blocks.

### Performance measures and exclusion criteria

Motion-tracking data were collected for the first 420 ms of the white mask, giving a total of 25 readings for each of the two sensors taken in every trial. The response for a single trial was calculated by computing the distances between pairs of readings and subtracting from them the distance between sensors collected when fingers were pinched together prior to stimulus presentation. The mean and standard deviation of the obtained values were then calculated. The mean distance was used as a measure of aperture for that particular trial. A large standard deviation was taken to indicate subject's noncompliance with instructions, for example if they accidentally closed the finger aperture long before the white mask disappeared from the screen. Those trials, where the variability lay beyond 2 standard deviations from subject variability mean, were excluded from analysis. In addition, those trials where response was smaller than 5 mm were also excluded. This exclusion criterion targeted those trials where subjects failed to pinch their fingers together prior to stimulus presentation.

### Visual appearance of numerical symbols

There is a possibility that the differential effects of numerical stimuli could have been driven by some latent visual features. Consequently, it would be useful to obtain a measure of similarity for visual appearance of stimuli. The locally linear embedding algorithm (Roweis & Saul, 2000) was used in order to fulfil this task. The algorithm reveals the latent structural similarity of objects via their projection onto a manifold of a lower dimensionality while preserving nonlinear relations of the original manifold. The relative separation between projections would indicate similarity/dissimilarity between items. In order to ensure that the original manifold was well sampled, the vectors encoding the images of numerical symbols were complemented with randomly generated vectors. These random vectors were obtained from the original images by random perturbation of the position of each pixel. Because numerical symbols were of five different sizes, the number of grey pixels used to draw numerical shape differed for the original images. To account for this, five groups of 200 random vectors were generated, a total of 1,000 vectors.

**Figure 2.**
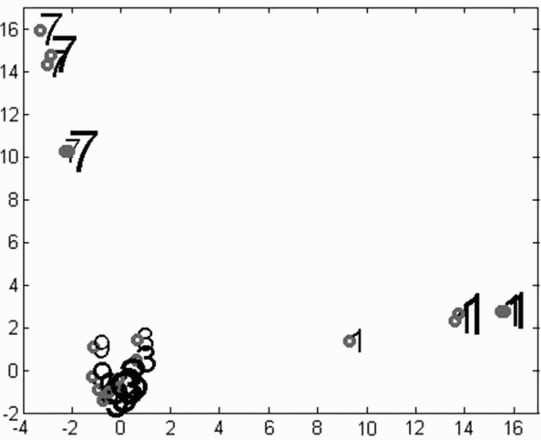
2-D projection of numerical magnitudes onto a manifold of a lower dimensionality using an locally linear embedding algorithm. The distance between data points indicates their structural similarity: The closer the data points the more similar they are. The sizes of the font for numerical symbols indicate the levels of size.

The two-dimensional projection accounting for most variance onto the new manifold is shown in Figure 2. The analysis shows a relative similarity between 3 and 9 as opposed to 1 and 7.

### Data analysis

Data were analysed in two steps. Firstly, the analysis of variance (ANOVA) was used as a filter to separate factors and interactions that significantly affected performance from the nonsignificant ones. The ANOVA analysis was run on the subjects' means, calculated for each condition, with array, size and number as within-subject factors and feedback (OL vs. CL) as a between-subject factor. Secondly, significant factors and interactions from ANOVA results were entered as predictors in a more detailed regression analysis of parametric effects. Linear regression models were fitted to the data for each subject independently. A subject-by-subject regression analysis arguably provides a more accurate estimate for the parametric effects than the group-level trend analysis, because, due to averaging artefacts, the group-level trend may be nonrepresentative of the individual functions that map from experimentally controlled variables into behaviour (Estes, 1956). The obtained samples of beta-values for each predictor were tested against zero. A visual inspection of the beta-value distributions indicated regular deviations from normality; therefore, a more robust Wilcoxon sign-rank test was adapted to determine whether a beta-value sample comes from a distribution with median equal to zero at the significance level of *p =* .05. The continuity of the response change between different levels of a variable of interest was evaluated using a paired *t* test on the subject means for those levels obtained after collapsing data across other experimental factors.

Because the influence of task-irrelevant numerical magnitude on aperture scaling is of a primary interest for this study, the regression analysis of number-related effects was run separately from the analysis of other factors. In addition to the above-described routines, the *t* test analysis of the data partitioned into small (1 and 3) and large (7 and 9) magnitude groups was also run. Given that a significant difference between two groups creates an impression of pseudoparametric mapping (e.g., Fischer, 2003; Lindeman et al., 2007), the findings of this sort have previously been used to argue for the common metric between number and space.

## Results

### Outliers

On the basis of the exclusion criteria defined above, 113 trials out of 6,400 (1.8%) were excluded in the OL condition and 71 trials out of 6,400 (1.1%) in the CL condition.

### Nonindependence of estimates

Prior to statistical analysis of the effects of experimental factors, the issue of nonindependence of responses should be addressed. Motor memory appears to play an important role in movement planning. Converging evidence suggests that the motor system tends to recycle the memory traces of previous responses, resulting in a systematic fluctuation in the variability (Diedrichsen, White, Newman, & Lally, 2010; Johansson & Westling, 1988; Slifkin & Newell, 1998). The present data also showed a considerable degree of the autocorrelation in responses for neighbouring trials, which gradually decreased as the lag between trials increased. The mean Pearson correlation for adjacent trials was .51 for the OL condition and .34 for the CL condition (*p* < .001). The influence of preceding trials was partialled out from the responses by regressing them on the responses from the previous trial. The first trials in the block, for which there was no preceding trial, were excluded from the analysis. In order to preserve between-subject variability for the following analyses, predicted variance was subtracted without centring the data—that is, the grand mean of subject responses after subtraction was equal to the grand mean of the original data.

### Full ANOVA analysis

The ANOVA analysis showed that all three within-subject main effects were reliably significant [array: *F*(1, 38) *=* 73.70, *p* < .001; size: *F*(4, 152) *=* 129.96, *p* < .001; number: *F*(3, 114) *=* 13.91, *p* < .001]. Among interactions, that of array and size reached significance level, *F*(4, 152) *=* 4.63, *p =* .005, showing that estimates for the small array tended to grow faster with the size of the target circle, as well as the interaction of all experimental factors, *F*(12, 456) *=* 2.60, *p* < .01. Given these results, the effects of array and size, their interaction, and the effect of number were analysed further using a linear regression technique.

### The effect of stimulus size and illusion

**Figure 3.**
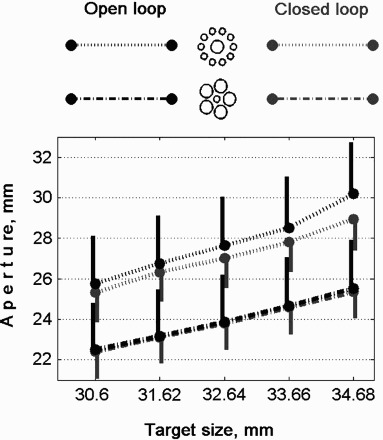
Manual estimates as a function of the array of surrounding circles and the size of the target circle size. Bars represent the standard error of subjects' mean responses.

The mean responses for size and array are shown in Figure 3. The regression model with array, size, and their interaction term as predictors together explained 21% of variance in the OL condition and 28% in the CL condition, suggesting considerable variability in individual responses. Although the median intercepts of the fitting models were slightly greater than zero (OL: 3.7 mm, CL: 4.38 mm), the analysis did not show that the difference was reliable, *p* > .10.

The betas for array were significantly greater than zero for both feedback conditions (both *p* < .001, *z* score approximation > 3.92). In other words, subjects provided smaller estimates when the target circle was surrounded by large circles than when it was surrounded with large circles. This replicates previous findings showing that the manual estimates are affected by the Titchener illusion (Amazeen & DaSilva, 2005; Haffenden & Goodale, 1998). The estimated median size of illusion was 2.7 and 3.2 mm for the OL and CL conditions, respectively

The betas for size also deviated from zero significantly for each feedback condition (*z =* 3.92, *p* < .001) showing that, despite great response variability, estimates monotonically increased with the size of target circle. The median increase in estimates for an increase of 1 mm in size were .81 mm and .77 mm for the OL and CL conditions, respectively. The paired *t* test on the means for different levels of size collapsed across other conditions showed that each level differed significantly from any other level, even an adjacent one, all *p* < .005 (Bonferroni-corrected for 10 comparisons). It should be noted that the grand average of standard deviations calculated for each condition and subject separately was 3.75 mm for OL and 2.8 mm for CL. If one is to take these values as a measure of discriminability for manual estimates, then it means that subjects demonstrated a monotonic increase in the estimates that is well below the discrimination threshold.

There was a significant or near-significant correlation between betas for size and array at the within-subject level for OL (Spearman rank correlation: *r =* .63, *p =* .003) and CL (*r =* .43, *p =* .058) condition, respectively, showing that the gain in response to the change in one of the stimulus parameters was proportional to the gain in response to the change in the other.

The interaction between array and size was significant only in the OL condition (*z =* 2.58, *p =* .01, CL: *z =* 1.61, *p =* .11). For OL, the gain rate for the small array was approximately 0.4 mm greater than that for the big array—that is, 1.08 and 0.65 mm, respectively (for CL, 0.86 and 0.75). None of the comparisons between betas for the two feedback conditions, including that for betas of the interaction term (rank-sum test for independent groups), was significant, suggesting that under two different feedback policies the subject exploited similar metrics.

### Analysis of the effect of number

**Figure 4.**
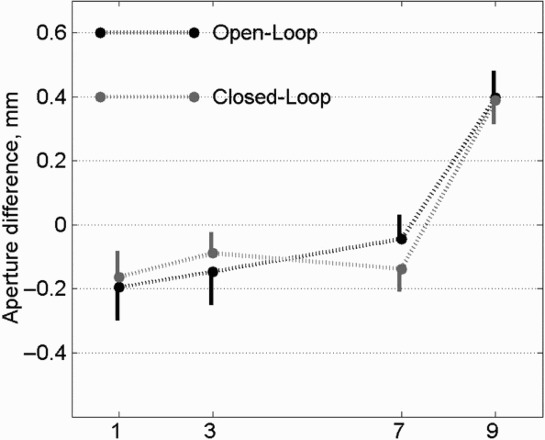
The means of aperture differences for each number, obtained after subtraction of variance predicted by size and array. The bars show the standard error of the subjects' mean.

Prior to the regression analysis of the effect of number, the variances explained by array, size, and their interaction were removed from the data. The mean results are shown in Figure 4. The residuals were then fitted with magnitude of number. Even though the beta-values significantly deviated from zero [OL: *p* < .005, median β *=* .064 (±.024) mm per unit magnitude; CL: *p* < .01, β *=* .042 (±.057)], the obtained *R*^2^ were very small (≤.003). The inclusion of squared and cubic terms (these terms were either significant or near significant for the group-level trend analysis) improved the predictive power of the models, *R*^2^ *=* .008 and .016 for OL and CL conditions, respectively. However, the improvement of the fit was at the expense of the significance of the linear term in both OL and CL conditions, *p* > .12.

The paired *t* test on the means for numbers collapsed across other conditions showed that the effect of number was predominantly driven by a larger aperture in responses for 9 than for other numbers (all *p* < .05, corrected for 6 comparisons for both OL and CL condition; other comparisons, *ns*, uncorrected). Following common practice (e.g., Fischer, 2003), the *t* test analysis was repeated for the data partitioned into small (1 and 3) and large (7 and 9) magnitude groups. The *t* test showed that the difference between two groups was statistically significant [OL condition: *t*(19) *=* 4.07, *p* < .002; CL condition: *t*(19) *=* 2.90, *p* < .01], suggesting that this sort of grouping does not reveal the real differences between magnitudes.

## Discussion

The hypothesis that number and space share a common metric with respect to action predicts that there is a structural similarity of their scales. The present study tested this prediction with a manual estimation paradigm. The estimates were provided in the context of the Titchener illusion. Subjects were required to scale the aperture between the index finger and the thumb in accordance with the size of presented stimuli either with or without visual feedback. The study replicated the findings from the previous reports showing that manual estimates reflect changes in the physical size of the target circle and are affected by the illusionary context (Amazeen & DaSilva, 2005; Haffenden & Goodale, 1998). Responses were more accurate for the closed-loop condition than for the open-loop condition reflecting the fact that the availability of visual feedback allowed for a better correction of the error (Woodworth, 1899).

The novelty of the present results relates to the two critical manipulations with the stimuli. First, manual estimation was found to reflect fine-grained changes in target stimuli. Despite the considerable variability of responding, average estimates showed a monotonic increase comparable with the objective increase in the stimulus size. These findings were supplementary to a more critical finding of a small but statistically reliable group-level effect of the magnitude of a task-irrelevant number presented inside the target circle. Subjects tended to provide a greater estimate if numerical magnitude was large, regardless of feedback availability. However, unlike the effect of the target stimulus size, the effect of number was nonparametric and was largely driven by a categorical distinction between the largest number in the range (9) and all others. The other “large” number (7) did not differ from “small” numbers even for uncorrected comparisons. This is unlikely to be explained by a relatively minor effect size for number in the presence of high variability. First, the differences between two adjacent sizes of target were also considerably smaller than the average standard deviation of the estimates. This fact did not prevent the estimates for the size from showing, on average, a reliable parametric increase. Second, the paired *t* test of number effect was run on the residuals obtained by subtracting the effect of all other factors, including the differences in individual grand means. Consequently, the variability in the data was substantially reduced, thereby making it easier to detect subtle effects. Meanwhile, the considerable variability in responses may explain why the presence of visual feedback did not eliminate the effect of the contextual information as may be expected on the basis of existing literature (Andres et al., 2008; Bruno & Franz, 2009; Glover, 2004). Given that the variability of responses marks a limit for control efficiency, the effects that are well below this threshold may be insensitive to the control mechanisms.

A separate analysis also showed that categorical experimental designs with group numbers as either large or small (e.g., Fischer, 2003; Lindeman et al., 2007) may not be informative about the relationship between number and space. Whereas the significant difference between two groups may create an impression of parametric mapping, the regression analysis and the number-by-number paired comparison suggest that this sort of grouping may obscure the real differences between magnitudes and is therefore insufficient to demonstrate a common scale.

An analysis was performed in order to establish whether the effect of number could be driven by some latent factors originating in the visual appearance of numerical symbol. A local linear embedding algorithm (Roweis & Saul, 2000) was used to project numerical symbols onto a manifold of a lower dimensionality. A close distance on the projected manifold indicates the visual similarity between items across different dimensions. This analysis is of a particular importance given the findings from a pointing study (Ishihara et al., 2006) showing that the number 7 does not gradually map onto space. Because the effect of this number was similar to the effect of 1, Ishihara et al. (2006) argued that this may be due to the visual similarity between 1 and 7. The analysis of the visual features indicated a relatively small similarity between these two numbers in the present study. It was found that the average distance between 9 and 3 was considerably smaller than the distance between them and other numbers. If the visual properties of numerical symbols were a critical factor, then one would expect that responses for 3, given its visual similarity to 9, would also be greater than responses for 1 and 7. This was, however, not the case.

The question remains why task-irrelevant numerical information affects motor performance despite the fact that the scales for number and space dissociate. One possibility is that they dissociate because the effects of number and size are constrained in different ways. Manual representations of variable target size may be strictly determined by the perceived size of the stimuli, whereas numerical magnitudes may create an imaginary context (De Hevia, Girelli, Bricolo, & Vallar, 2008) directly affecting movement execution. Although this may be the case, one can note that manual estimation is not just a report of the perceived stimulus size. Amazeen and DaSilva (2005) were the first who argued that this view would be too simplistic. They showed that the illusionary effects are stronger for manual estimation than for perceptual reports. Their analysis also showed that percepts used for the perceptual reports and for manual estimation are at least in part independent, despite the fact that both are affected by illusion. The present results identify two additional points of the deviation of the manual estimate from being a simple report of the perceived size. These deviations seem to occur at the stage of mapping a percept into a motor response. First, responses showed a relatively high degree of autocorrelation. This suggests that the response in manual estimation is coded in two complementary reference frames: One is determined by the actual size of the stimulus; the other is determined by the memory traces of previous motor commands. A considerably weaker autocorrelation in the closed-loop condition also suggests that the functional role of the visual feedback is monitoring performance not simply to decrease variability, but also to transform routinely repeating behaviours, based on prior motor memories, into an object-oriented performance. Second, there was a correlation between beta values for array and size at the within-subject level or, in other words, the gain in response to the change in surrounding array was proportional to the gain in response to change in the target circle. Here again the correlation was weaker for the closed-loop condition. Given that the open- and closed-loop conditions were identical in respect to the perceptual processing of stimuli, this modulation of the relations between gains suggests a nonperceptual origin for the latter.

An alternative explanation for the effect of number on manual estimates is that the number magnitude interacted with categorical representations of space. This type of representation can provide contextual cues for movement planning (Glover, 2004), but does not represent the proper metric for action. This view would relate the effect of number to the effects of the other types of symbolic stimuli that bear implicit magnitude semantics. Current theories of motor control describe mechanisms that can enable such interaction. According to these theories, sensorimotor processes are formally equivalent to a decision under uncertainty (Trommershauser, Maloney, & Landy, 2008), because the motor system constantly faces a selection from an unlimited number of options while executing a single movement. It is now believed that the motor system utilizes contextual and memory-based information (priors) in order to constrain the decision space and simultaneously counteract the inherent noise in sensory and motor signals (Kording & Wolpert, 2006). This is consistent with the idea that, according to Tzelgov et al. (1992), the origin of categorical representations of numbers is an everyday experience in which subjects consistently classify numbers as small and large. The retrieval of categorical values is relatively effortless, and therefore they can be relied on as long as a task does not require a fine-grained scale to address the problem.

The present findings support the view that representational models for numbers may assume different forms and may not necessarily be continuous. Despite earlier claims that there is a unique format for number representations, such as the mental number line (Dehaene, 2003), more recent findings challenge this view (Gevers et al., 2010; Van Dijck, Gevers, & Fias, 2009). The proposal that there are many different kinds of representations for numerical magnitude is supported by the evidence that the representational models for numbers appear to adapt easily to the requirements of the task (Karolis, Iuculano, & Butterworth, 2011; Van Dijck et al., 2009). A switch from continuous mapping onto space to a categorical one may be elicited by asking subjects to perform magnitude comparison task instead of parity judgements (Gevers et al., 2006).

What are the principles for categorization of numbers into small and large? It has been argued that number 5 has a special role as a natural borderline between sets of small and large numbers for the range 1–9 (Link, 1990; Tzelgov et al., 1992). This categorization, however, has been discussed in connection with the magnitude comparison paradigm and may be triggered by specific features of the task. This is often elicited by explicit instructions such as “press left key if number is < 5 and the right key if the number is > 5”. When the comparison is between any two numbers rather than between a number and a standard, such categorization may still have behavioural relevance if speeded judgements are required. As Link (1990) suggests, magnitude judgements may be analysed in probabilistic terms: The probabilities of responding smaller are not equal between numbers. It is more likely to respond “smaller” for numbers 1–4 than for numbers 5–9 with the point of equal objective probability for responding “smaller” and “larger” centred on 5. Consequently, the model that categorizes number in this way optimizes behaviour and increases chances to respond correctly at a rapid rate.

Such a categorical model does not automatically generalize to other tasks. For example, under different experimental settings, when subjects are required to enumerate items after a brief exposure, the number 4 may be considered as a borderline between small and large numbers—the small set is also known as a subitizing range (Trick & Pylyshyn, 1994). The task presented in this study did not have time constraints, nor did it require a selection between alternatives, and, therefore, the categorization of numbers could be principally different from that in any of the above examples. The results suggest that subjects implicitly categorized 9 as large number and all smaller numbers as small. Even the presence of a gap in the stimulus range, through omitting the number 5, did not prompt a subject to use the categorization with respect to 5. The question is whether the observed categorization of 9 as a large number and the rest as small can be psychologically relevant. A tentative answer may be as follows. One of the most widely known results in cognitive science is that the ability to represent differences between items along a unidimensional continuum is limited to approximately 7 items (Miller, 1956). This fact has been related to a limited cognitive capacity to transmit information. With respect to measurement theory (Stevens, 1968), this is equivalent to the limited capacity of assigning a number to a stimulus magnitude. Consequently, these limits may suggest a naturalistic model for a categorization into small and large sets with 7 rather than 5 completing the set of small numbers. One could speculate that the present results represent the first support for such categorization.

In conclusion, it should be noted that it is of a particular importance for the hypothesis of the shared metrics that numerical magnitudes may interact with the spatial movement parameters in different ways. Previous research on the effect of number may underestimate the fact that different types of motor behaviour may rely on different computations (but see Andres et al., 2008). For example, the type of movement investigated in the present study is a nonrapid and imitated movement, rather than one that aims at getting in contact with an object (see also Andres et al., 2004, and Moretto & di Pellegrino, 2008). Therefore, it may not be surprising that the parametric effect here was lacking, as imitated and actual motor responses may rely on the different neural computations and different representations of space (Carey, Dijkerman, Murphy, Goodale, & Milner, 2006). The present finding of the categorical effect of number on the nonrapid motor responses does not in itself refute the hypothesis of shared metrics for number and space. However, these findings stress that without a meticulous scale analysis as a starting point of inquiry, the nature of the number–space interaction may remain indeterminable.
